# Metal–Analyte Interactions in the Forensic Detection of Psychoactive Compounds: A Narrative Review

**DOI:** 10.3390/molecules31193394

**Published:** 2026-09-24

**Authors:** Dušan Dimić

**Affiliations:** Faculty of Physical Chemistry, University of Belgrade, Studentski trg 12-16, 11000 Belgrade, Serbia; ddimic@ffh.bg.ac.rs

**Keywords:** psychoactive compounds, forensic analysis, metal interactions, metal-based sensors, SERS, luminescence, electrochemical sensing

## Abstract

The continuous diversification of psychoactive substances and the complexity of forensic samples create an increasing demand for rapid, sensitive, and portable analytical approaches complementary to conventional chromatographic and mass-spectrometric methods. Interactions between psychoactive compounds and metals offer versatile detection mechanisms by converting molecular recognition, adsorption, redox reactions, and interfacial processes into measurable analytical signals. This narrative review summarizes the principal applications of metal–analyte interactions in the forensic analysis of psychoactive compounds, ranging from classical presumptive color tests and spectrophotometric and luminescence methods to metal-based nanoprobes, surface-enhanced Raman spectroscopy (SERS), and electrochemical sensors. This review emphasizes the analytical role of metals rather than individual classes of psychoactive substances. The metal-containing species and materials considered include free metal ions, coordination complexes, and redox-active species, as well as metallic nanoparticles, quantum dots, metal oxides, metal–organic frameworks, and metal-containing components of modified electrode surfaces, which participate in analyte recognition and signal generation through coordination, ion association, redox processes, adsorption, charge transfer, plasmonic enhancement, and electrocatalysis. Recent developments demonstrate remarkable improvements in sensitivity and compatibility with portable instrumentation. However, translation into routine forensic practice remains limited by matrix effects, selectivity, reproducibility, and insufficient validation with authentic samples. Understanding the underlying metal–analyte interactions is therefore essential for transforming promising sensing concepts into reliable forensic analytical platforms.

## 1. Introduction

The continuous diversification of psychoactive substances represents an important challenge for modern forensic analysis [1,2,3]. In addition to conventional illicit drugs, forensic laboratories increasingly encounter synthetic stimulants, opioids, hallucinogens, dissociatives, and other emerging psychoactive substances, frequently accompanied by adulterants, cutting agents, pharmaceutical ingredients, and structurally related analogs [4,5]. Reliable identification generally relies on chromatographic and spectrometric techniques, particularly gas or liquid chromatography coupled with mass spectrometry [6,7]. Although these methods provide high selectivity and are indispensable for confirmatory analysis, considerable interest remains in rapid, inexpensive, and potentially portable approaches that can facilitate preliminary identification, screening, and on-site analysis of seized materials and biological samples [8]. In this context, interactions between psychoactive compounds and metals provide particularly versatile mechanisms for converting molecular recognition into measurable analytical signals [9,10].

The analytical importance of metals arises from the chemical diversity of both psychoactive compounds and metal-containing systems. Many psychoactive substances contain nitrogen-, oxygen-, and, less frequently, sulfur-containing functional groups capable of coordination, ion association, redox reactions, or adsorption onto metallic surfaces [11,12,13,14,15]. Depending on molecular structure and experimental conditions, these interactions may involve amino, heterocyclic nitrogen, carbonyl, hydroxyl, ether, or sulfur-containing groups [16,17,18,19]. Metals, in turn, can participate as free ions, coordination complexes, redox-active species, metallic nanoparticles, metal oxides, metal–organic frameworks, semiconductor materials, or components of modified electrode surfaces. Consequently, the term metal–drug interaction encompasses considerably more than classical coordination-complex formation. It includes a broad range of interfacial, electronic, catalytic, and supramolecular phenomena that can be exploited for analytical purposes. Figure 1 shows the most important groups of methods discussed in this review.

Some of the earliest forensic applications of these interactions are found in presumptive color tests [20]. Formation of colored metal–drug species or ion-association products provides an immediate visual response and has enabled rapid screening of controlled substances for decades. The well-known interaction of cocaine with cobalt thiocyanate illustrates both the utility and limitations of this approach: a simple metal-based reaction provides rapid presumptive identification, but competing interactions with structurally related compounds and adulterants can compromise selectivity [21]. More sophisticated spectrophotometric and luminescence methods exploit the same fundamental chemical versatility through metal-mediated oxidation, formation of absorbing coordination or ion-association species, modulation of fluorescence, sensitization of lanthanide emission, and generation of chemiluminescence [22,23,24,25]. Metal ions, therefore, serve not merely as recognition centers but also as redox mediators and signal-generating species, allowing their interaction with a psychoactive compound to be translated into an optical response.

The development of nanostructured materials has substantially expanded the analytical roles of metals. Plasmonic Au and Ag nanostructures form the basis of surface-enhanced Raman spectroscopy (SERS), in which adsorption of an analyte in proximity to the metallic surface results in substantial enhancement of its characteristic Raman spectrum [26,27,28]. While electromagnetic enhancement associated with localized surface plasmon resonance generally dominates, chemical interactions and charge-transfer processes between the analyte and metal surface can additionally affect spectral intensity and profile. Such systems combine the molecular specificity of vibrational spectroscopy with high sensitivity and have enabled the detection of psychoactive compounds in matrices of direct forensic relevance, including seized materials, oral fluid, urine, and latent fingerprints [28,29,30,31]. The role of the metal in these systems is therefore fundamentally different from that in conventional color tests: rather than necessarily forming a new coordination species, the metal provides an interfacial environment that simultaneously concentrates the analyte and amplifies its spectroscopic signature.

Metal-containing materials have likewise become important components of electrochemical sensing platforms [32,33,34,35]. Coordination complexes can provide selective interaction sites and reversible redox mediators, whereas metallic nanoparticles, metal oxides, mixed-metal materials, and metal–organic frameworks can enhance electron transfer, increase electroactive surface area, promote analyte adsorption, and catalyze oxidation or reduction processes. These properties are particularly useful for psychoactive substances whose electrochemical responses at conventional electrodes may require relatively high potentials or may be affected by overlapping signals and electrode fouling. Direct coordination of the analyte with an electroactive metal center, metal-mediated redox transformations, and electrocatalysis at nanostructured metal-containing surfaces consequently represent mechanistically distinct routes toward sensitive detection. The compatibility of these materials with screen-printed electrodes and portable potentiostats further increases their potential for decentralized forensic screening.

Despite extensive development, metal-based methods differ considerably in their analytical maturity and forensic relevance. Many sensors achieve impressive detection limits under optimized laboratory conditions, yet fewer have been evaluated using authentic seized materials, complex biological specimens, common adulterants, or structurally related psychoactive compounds. Analytical sensitivity alone, often presented in the literature, is not sufficient for practical applications. Selectivity, reproducibility, matrix tolerance, robustness, portability, speed, and the ability to discriminate related substances are equally important considerations. This distinction is particularly relevant for nanomaterial-based SERS and electrochemical sensing, where a rapidly expanding literature contains numerous variations in substrate architecture and composition but comparatively fewer studies addressing implementation under realistic forensic conditions.

Previous reviews in this area have generally focused on individual analytical approaches, including SERS, electrochemical sensing, nanomaterial-based sensors, or conventional presumptive tests [20,26,28,33]. Although these contributions provide detailed assessments of particular analytical platforms, they have given comparatively less attention to the common chemical role of metals across these otherwise distinct methodologies. This review critically examines the use of metal interactions to detect and analyze psychoactive compounds, with particular emphasis on the underlying chemical mechanisms and forensic significance. In the context of this review, the term “psychoactive compounds” is used broadly to include controlled drugs, new psychoactive substances, psychoactive pharmaceuticals, and relevant metabolites that may be encountered in forensic drug analysis or forensic toxicology. The primary emphasis is on methods applicable to screening or analyzing seized materials and biological specimens. Studies developed primarily for pharmaceutical or environmental analysis are included selectively when they provide mechanistic information on metal–analyte interactions or demonstrate analytical principles that could potentially be transferred to forensic applications. Their inclusion should therefore not be interpreted as evidence of forensic validation, and this review distinguishes proof-of-concept analytical studies from methods evaluated in forensically relevant matrices.

This review discusses classical presumptive tests, spectrophotometric and luminescence methods, SERS using plasmonic metal substrates, and electrochemical sensing based on metal complexes and metal-containing materials. Rather than being restricted to individual structural classes of psychoactive compounds, the literature is organized by the metal’s analytical role, including molecular recognition and coordination, ion association, redox mediation, signal generation, plasmonic enhancement, adsorption, and electrocatalysis. Given the particularly extensive literature on SERS and electrochemical sensors, representative studies are emphasized to illustrate mechanistically distinct interactions, analytical platforms, sample matrices, and forensic applications rather than to provide an exhaustive catalog of reported systems. This approach connects the fundamental chemistry of metal–analyte interactions with their practical analytical consequences while identifying opportunities and remaining challenges in translating metal-based detection strategies into reliable tools for forensic analysis.

## 2. Colorimetric Presumptive Tests Based on Metal Complexation

One of the oldest and most widely used forensic applications of coordination chemistry is the development of presumptive color tests [20]. These methods rely on the rapid formation of colored coordination compounds, whose appearance serves as an initial indication of the presence of a particular class of psychoactive substances.

Among the compounds discussed in this review, cocaine represents the best example of the successful application of metal complexation in forensic analysis. The well-known Scott test relies on the reaction of cocaine with cobalt(II) thiocyanate, producing an intense blue coordination compound that enables rapid field screening of cocaine-containing samples [9]. Structural investigations combining FTIR spectroscopy and density functional theory suggested that the observed color originates from the formation of the coordination compound [Co(COC)(NCS)(H_2_O)_3_]SCN, in which cocaine coordinates through a nitrogen atom and an ether oxygen atom, while electrochemical studies demonstrated that the relatively low stability constant of the complex explains the requirement for concentrated analyte solutions during forensic screening. The explanation based solely on FTIR spectra and DFT calculations should be taken with caution, as the crystallographic structure was not obtained, and the existence of other products cannot be excluded. These findings illustrate how structural studies can improve the mechanistic understanding of classical forensic color tests. Cobalt thiocyanate chemistry is not restricted to cocaine and has also been adapted for the presumptive identification of ketamine hydrochloride. Morris modified the conventional procedure by introducing a basification step, demonstrating that manipulation of protonation and extraction conditions can alter the selectivity of Co(II)-based color reactions toward different psychoactive compounds [36]. Despite their simplicity, Co(II)-based presumptive tests remain vulnerable to chemically unrelated interferents [37]. Notably, fentanyl has been reported to produce cocaine-like responses with aqueous cobalt thiocyanate and Young’s tests, while discrimination using Scott-type procedures was dependent on the amount of material tested. Such observations emphasize that visually interpreted metal–drug reactions remain presumptive and require subsequent confirmatory analysis.

The interactions of psychoactive compounds with metal ions and the formation of colored complexes can also be used to detect metals, which is applicable in analytical chemistry, although the actual forensic importance is limited. For example, phenothiazine derivatives form complexes with Pd(II), producing characteristic absorption bands around 460 and 540 nm that have been successfully applied for spectrophotometric palladium determination [38]. A similar phenomenon was described for promazine, as it readily forms ion-association compounds with ferricyanide, ammonium reineckate, cobaltinitrite, and several transition-metal halide and thiocyanate complexes, allowing sensitive spectrophotometric determination of both the drug and metal ions [39].

Metal-based colorimetric tests offer important advantages in simplicity, rapidity, low cost, and minimal instrumentation, making them particularly suitable for field screening. Their principal limitation is selectivity, since visually similar responses may be produced by structurally related compounds or unrelated interferents, while sensitivity is generally lower than that of instrumental approaches. Their greatest forensic value therefore remains as inexpensive presumptive tests rather than methods for definitive identification.

## 3. Spectrophotometric and Luminescent Methods Based on Metal Interactions

Fluorescence methods are a promising way of detecting trace amounts of psychoactive compounds due to their rapid response time, straightforward use, and high sensitivity [40]. Metal ions and metal-containing nanomaterials provide several complementary routes for optically determining psychoactive compounds. Rather than relying on a single coordination reaction, reported methods exploit changes in metal oxidation state, formation of colored ion-association species, modulation of metal-centered or nanoparticle-based emission, and generation or enhancement of chemiluminescence. These mechanisms convert the interaction of a psychoactive molecule with a metal-containing reagent into an absorbance or emission signal and have been applied to pharmaceutical preparations, seized-drug screening, and biological matrices.

As mentioned in the review paper by Kyndait and coworkers [41], luminescent sensors based on transition-metal complexes offer long emission lifetimes due to mixed metal–ligand states; self-quenching is minimal because absorption and emission are separated; and these systems often exhibit high quantum yields.

### 3.1. Metal-Mediated Oxidation and Formation of Absorbing or Emissive Products

One of the most established approaches is based on oxidation of the analyte by a metal ion in a high oxidation state. In these systems, the concentration of the psychoactive compound is determined indirectly by measuring either the amount of reduced metal species formed during analyte oxidation or the amount of unreacted oxidant remaining after the reaction. Morphine, for example, can be oxidized by Ce(IV) in an acidic medium, producing fluorescent Ce(III) [25]. The specificity of this procedure is limited, as metal-ion reduction can proceed in the presence of other compounds. Measurement of the Ce(III) emission at 350 nm, therefore, provides a spectrofluorimetric response proportional to the amount of oxidized drug. The same reaction can be monitored spectrophotometrically by adding an excess of Ce(IV), allowing the unreacted oxidant to react with methyl orange, and measuring the remaining dye absorbance at 510 nm. Thus, fluorescence and absorbance measurements represent two analytical readouts of the same underlying redox process. The methods were applied to pharmaceutical formulations and biological fluids, which is particularly relevant because morphine may be encountered both as an administered analgesic and as a product of heroin metabolism. A similar method was proposed by Mohamed and coworkers for the oxidation of chlorpromazine HCl, thioridazine HCl, clomipramine HCl, imipramine HCl, and desipramine HCl by Ce(IV) in sulfuric acid [42].

The reduction of Cu(II) to Cu(I) and subsequent complexation with the BODIPY-Phen ligand, in which the 1,10-phenanthroline moiety is connected to the BODIPY dye, is proposed as a novel method for the determination of ketamine and scopolamine [43]. In this system, complexation quenches the ligand’s fluorescence and produces a visible color change, making this test suitable for quantifying compounds in spiked drinks. The detection limits for both drugs were in the micromolar range. Other amines produced a medium response, while methylone and diazepam showed no response. A similar meso-4-nitrophenyl derivative of BODIPY was successfully applied for the on-site detection of synthetic cathinones, ketamine, and scopolamine in beverages [44]. The electron paramagnetic resonance results showed that the color change resulted from radical formation. Both methods rely on the fact that these psychoactive compounds can reduce Cu(II) to Cu(I) [45]. The same principle was relevant for metal-based presumptive testing developed for synthetic cathinones using Cu(II)–neocuproine. In this system, electron-rich cathinone derivatives reduce Cu(II) to Cu(I), followed by formation of a yellow-orange Cu(I)–neocuproine complex with an absorption maximum at approximately 453 nm [45]. The presumptive test produced positive responses for 39 of 44 investigated cathinones and retained its characteristic response in samples containing glucose or caffeine, as well as in mixtures of synthetic cathinones. The absence of color change in samples containing amphetamines and other illicit compounds showed selectivity toward cathinones. The method, therefore, illustrates how metal-mediated redox chemistry, followed by coordination of the reduced metal ion, can provide a class-based presumptive response to emerging psychoactive substances.

Phenothiazine drugs are especially suitable for oxidative spectrophotometric assays because their electron-rich sulfur- and nitrogen-containing aromatic systems readily yield intensely colored oxidation products. Promazine is oxidized by Cr(VI) in an acidic medium to form a colored species that can be used directly for spectrophotometric determination [46]. In parallel, dichromate forms a sparingly soluble ion-association compound with protonated promazine, which can be extracted into ethyl acetate and measured in the organic phase. This method consequently combines two effects: oxidation of the phenothiazine chromophore and association of the cationic drug with a metal-containing anion.

A related, but more sensitive, redox strategy uses the analyte to modulate a chemiluminescent reaction. Papaverine enhances the weak emission generated by the acidic Ce(IV)–sulfite system [23]. The gradual formation of fluorescent Ce(III) demonstrates that papaverine is oxidized by Ce(IV), producing a papaverine-derived radical intermediate that increases the chemiluminescence of the sulfite–Ce(IV) reaction. Coupling this process with flow-injection analysis provided a rapid method applicable to pharmaceutical preparations and biological fluids, with a submicromolar detection limit. These examples show that metal-mediated oxidation can be monitored by absorbance, fluorescence, or chemiluminescence, depending on whether the analytically useful species is the oxidized drug, the reduced metal ion, the residual oxidant, or an electronically excited reaction product.

### 3.2. Ion Association, Complex Formation, and Extractive Spectrophotometry

The second group of methods is based on the formation of colored coordination or ion-association species. Protonated psychoactive amines can associate with negatively charged metal complexes, producing species with altered solubility and characteristic optical properties. The analytical response may be measured directly in solution or after liquid–liquid extraction, which simultaneously separates the analyte from interfering matrix components and concentrates the colored product. One example includes the formation of the Eu(III)-bromazepam complex, as the presence of the psychoactive compound enhances the luminescence intensity of Eu(III) when excited at 390 nm (Table 1) [47].

The quantitative detection limit for bromazepam in acetonitrile was 9.2 × 10^−9^ M. The authors used only a UV–VIS spectrophotometer and a spectrofluorometer to discuss complex formation; therefore, other types of interactions cannot be excluded.

This principle is especially important for phenothiazines. Promazine, promethazine, and chlorpromazine form ion associates with metal-containing counterions such as cobaltinitrite or ferricyanide [39]. Elemental analysis, FTIR, and thermogravimetric measurements were used to characterize these ion associates and verify their compositions. Such reactions have been employed for spectrophotometric determination and, in some cases, for indirect determination of the metal remaining in or transferred to the analytical phase. The main analytical role of the metal species is therefore not necessarily the formation of a classical inner-sphere coordination compound. Instead, the metal complex acts as a large, optically active counterion that selectively associates with the protonated nitrogen-containing drug.

Direct chelation can also generate analytically useful absorption changes. Ephedrine forms an extractable complex with Co(II), and the resulting species can be determined by UV–Vis spectroscopy after transfer into chloroform [24]. Because the product was not comprehensively characterized, the complexation can only be proposed based on changes in the absorption spectrum. The response was used to quantify ephedrine hydrochloride in pharmaceutical samples, with complex formation controlled by pH, cobalt concentration, reaction time, and extraction conditions. Although such procedures were developed primarily for pharmaceutical analysis, the same combination of selective complexation and phase separation may be useful for preliminary analysis of psychoactive compounds in comparatively simple forensic samples.

Suppression of phosphorescence can be an alternative pathway for sensor development. In the paper by Wang and coworkers, an iridium-containing complex surrounded by bipyridine and phenanthroline was used for the detection of γ-hydroxybutyrate (GHB) [48]. This complex had a large Stokes shift between excitation and phosphorescent emission (300 nm and 570 nm) and a long lifetime. When GHB was present at a concentration of 12.4 mM, the phosphorescence intensity was reduced by 60%, which was attributed to binding-induced perturbation of the excited state of the complex. The same compound was also detectable by BODIPY-Fe(III), which allowed the production of a low-cost sensor and presumptive test, as shown in [49]. The authors concluded that adding GHB led to an interaction between the drug’s carboxyl group and the disruption of one interaction between the ligand and the central metal ion. This was observed through changes in absorbance maxima. Other commonly found drugs did not interfere with this process. The removal of the ligand was employed in the paper by Manez and coworkers [50] in which coumarine derivative was removed from Cu(II) quinoline complex, with the formation of direct Cu(II)-GHB interaction.

### 3.3. Fluorescence Quenching by Metal-Doped Quantum Dots

Metal-containing quantum dots (QDs) provide a different sensing mechanism in which recognition of the drug changes the emission of a nanocrystalline probe. QDs show size-tunable emissions, high quantum yields, and narrow emission lines, and have been applied recently for a variety of illicit drugs [52,68,69].

Mn-doped ZnS quantum dots coated with a molecularly imprinted polymer have been developed for selective cocaine recognition [51]. The imprinted cavities bind to cocaine and structurally related metabolites near the quantum-dot surface, causing concentration-dependent fluorescence quenching. The lack of comparable quenching with non-imprinted particles or unrelated drugs shows that selectivity arises primarily from molecular imprinting, while the Mn-doped ZnS core provides the luminescent signal. This approach was subsequently adapted for cocaine screening in oral fluid and serum [70]. The binding of cocaine to the imprinted layer surrounding the Mn-doped ZnS quantum dots decreased the fluorescence intensity, while sample dilution, centrifugation, or solid-phase extraction was required to limit matrix effects. The successful application of oral fluid and serum is particularly significant for forensic toxicology because these matrices contain proteins, salts, and endogenous fluorescent or quenching species that can strongly influence nanoparticle emission. The method illustrates a general division of functions in metal-based nanosensors: the polymeric surface provides molecular recognition, while the doped semiconductor serves as a sensitive optical transducer.

The complexity of nanoprobes extends beyond direct interaction between metals and psychoactive substances. In a novel system, the presence of Co(II) as a precursor for carbon dots synthesis allowed changes in recombination pathways through the reduction of non-radiative energy losses [52,71]. Adrenaline was added as a functional monomer on Co(II)-QD, allowing the formation of specific hydrogen bonds with methamphetamine, leading to increased sensitivity of the whole system towards this drug [52]. The authors postulated that the formation of a non-fluorescent complex between methamphetamine and the sensor is a consequence of the interaction of Co(II) with the amino group of the drug. This method had a detection limit of 23 nM of methamphetamine in simulated urine. A similar limit for methamphetamine detection was obtained for the system containing EDTA in the molecularly imprinted sensor [40]. In this case, specific binding resulted from interactions on the surface of the molecularly imprinted polymer; without it, the fluorescence enhancement of Cu(II)-EDTA quantum dots showed a similar response to other psychoactive compounds.

A more elaborate metal-containing fluorescence platform was developed for 3,4-methylenedioxymethamphetamine (MDMA) using antibody-functionalized AuZnFeSeS alloyed quantum dots coupled with NiCeFe_3_O_4_ magnetic nanoparticles [53]. Magnetic nanoparticles act as adsorbents, magnetically separating bound complexes [72]. The quantum dots served as fluorescence reporters, while the magnetic nanoparticles enhanced analyte capture and amplified the signal, producing an OFF–ON–OFF fluorescence response upon nanocomplex assembly and subsequent MDMA binding. Importantly, the NiCeFe_3_O_4_ component was essential for selectivity, as the antibody-functionalized quantum dots alone did not effectively discriminate MDMA from structurally related and potentially interfering compounds. The combined system achieved a detection limit of 0.02 nM (0.0046 ng mL^−1^). It was successfully applied to urine, illustrating how different metal-containing nanomaterials can be integrated to combine molecular recognition, signal amplification, and fluorescence transduction in a single platform.

### 3.4. Metal Nanocluster and Metal–Organic Framework Luminescence Modulation

Other metal-based probes operate through a “turn-on” mechanism, in which analyte binding increases emission rather than quenching it. A Cr(III)-based metal–organic framework was employed for dual visual and photoluminescent detection of morphine [54]. Adding morphine caused a blue shift in the emission maximum from 593 to 566 nm, a marked increase in photoluminescence intensity, and a visible color change from brown to yellow. The combined wavelength shift, emission enhancement, and naked-eye response enabled morphine detection in serum and urine at nanomolar concentrations. The rigid and porous MOF structure provides a confined recognition environment in which interaction with morphine modifies the electronic states responsible for emission.

A recent hybrid fluorescence platform combined levofloxacin-derived carbon dots with the Zr-based MOF UiO-66-NH_2_ for the detection of ketamine, morphine, and heroin in tap water and wastewater [55]. In this system, the porous MOF primarily served as an adsorption and preconcentration matrix, whereas the incorporated carbon dots provided the optical transduction. The composite showed markedly stronger fluorescence responses than either component alone and achieved sub-ng L^−1^ detection limits for all three drugs. Although the method was validated in tap water and wastewater rather than conventional forensic matrices, it illustrates how metal–organic frameworks can be coupled with fluorescent nanomaterials to integrate analyte enrichment and signal generation within a single sensing platform.

Fluorescence recovery has also been achieved using gold nanoparticles and an organic fluorophore [56]. Fluorescein emission is initially quenched by Au nanoparticles through nanometal surface energy transfer because the nanoparticle absorption overlaps the fluorescein emission. Morphine coordinates to the nanoparticle surface through its tertiary nitrogen and phenolic hydroxyl group, altering the fluorophore–nanoparticle interaction and restoring fluorescence. The sensing mechanism was proposed based on the transmission electron microscopy images and fluorescein lifetime. The requirement for both functional groups contributes to selectivity, and the same mechanism was incorporated into a paper-strip assay for morphine in sweat, urine, serum, and other biological fluids.

Lanthanide-doped nanoparticles provide another form of turn-on detection. Captopril-modified Y_2_O_3_:Eu^3+^ nanoparticles showed enhanced Eu(III)-centered emission at 612 nm in the presence of methamphetamine [57]. The increase was attributed to the interaction of methamphetamine with the modified nanoparticle surface, which altered the efficiency of excitation or nonradiative deactivation of the Eu^3+^ emitter. The method operated at biological pH and ambient temperature, supporting the potential use of rare-earth emission as a stable and spectrally narrow signal for stimulant detection.

Paria et al. developed mono-, bi-, and trimetallic Au-, AuAg-, and AuAgCd nanoclusters stabilized by lysozyme (Lyz), glutathione (GSH), or 3-mercaptopropionic acid (MPA) for fluorescence-based detection of amphetamine and morphine [58]. The addition of various metals to gold and silver nanoclusters induces fluorescence quenching and enhancement through various mechanisms [73]. The trimetallic AuAgCd-Lyz nanoclusters showed the strongest fluorescence turn-on response, outperforming the corresponding mono- and bimetallic systems as well as thiol-stabilized AuAgCd nanoclusters. This behavior was attributed to the relatively labile Au–amide interactions in the lysozyme-protected system, which facilitate analyte-induced structural rearrangement and modulation of metal-centered charge-transfer emission. Binding constants of 5.82 × 10^3^ and 7.89 × 10^3^ M^−1^ and LODs of 4.44 and 2.16 μM were obtained for amphetamine and morphine, respectively.

### 3.5. Formation of Analyte-Specific Photoluminescent Coordination Clusters

A particularly promising forensic strategy is based on the direct formation of emissive coordination products whose spectral profiles depend on the psychoactive ligand. Cu(I) iodide reacts with heterocyclic nitrogen-containing compounds to form photoluminescent clusters. In contrast, analogous complexes of several other metal ions and simple amines generally do not emit strongly in the visible region [59]. Cocaine, phencyclidine, benzylpiperazine, morphine, codeine, heroin, caffeine, and related substances produced distinguishable emission profiles after reaction with CuI. For example, the CuI–codeine product showed two emission maxima, whereas clusters formed with heroin and hydrocodone produced different major and minor bands. The method was able to distinguish cocaine from phencyclidine and benzylpiperazine, compounds for which conventional cobalt thiocyanate color testing may be insufficiently selective. Unlike a conventional color test that provides only a positive or negative visual response, the entire photoluminescence spectrum can function as a chemical fingerprint. Construction of a reference library of CuI–drug emission profiles could therefore support rapid presumptive classification of controlled substances and common cutting agents.

From an analytical perspective, spectrophotometric and luminescent metal-based methods combine relatively straightforward operation with high sensitivity, particularly when fluorescence or phosphorescence amplifies the signal. Their performance, however, may depend strongly on reaction conditions, probe composition, and sample matrix, while enhanced optical response does not necessarily imply molecular selectivity. Although these approaches can offer lower instrumental complexity than chromatographic–mass spectrometric methods, broader forensic application requires further validation with realistic forensic samples and relevant interfering substances.

## 4. Surface-Enhanced Raman Spectroscopy and Metal Interactions

Surface-enhanced Raman spectroscopy (SERS) is one of the most successful analytical applications of metal–drug interactions for detecting psychoactive compounds [74,75,76,77]. In conventional Raman spectroscopy, only a small fraction of incident photons undergoes inelastic scattering, which inherently limits sensitivity. In SERS, nanostructured plasmonic surfaces, such as those based on Ag or Au, can increase Raman scattering by several orders of magnitude. The dominant electromagnetic contribution originates from localized surface plasmon resonances, which generate strongly enhanced local electromagnetic fields near the metal surface, particularly at nanoscale junctions, sharp features, and interparticle gaps commonly referred to as “hot spots.” A second, usually smaller, chemical contribution can arise from adsorption-induced changes in molecular polarizability and charge-transfer interactions between the analyte and the metal surface. Consequently, SERS performance depends not only on the identity of the metal but also on particle size and morphology, surface roughness, aggregation state, excitation wavelength, and the adsorption geometry of the analyte [78,79]. Recent developments in engineered porous, patterned, and nanostructured plasmonic substrates have consequently focused on generating reproducible distributions of high-field regions while improving substrate stability and measurement-to-measurement reproducibility [26,80]. The importance of these chemical contributions has been demonstrated particularly clearly for amphetamine-type stimulants in the paper by Smeliková and coworkers [81]. Comparative studies of amphetamine, methamphetamine, and MDMA on Au and Ag surfaces showed that the resulting spectra cannot always be interpreted solely in terms of electromagnetic enhancement. DFT-supported analysis indicated that drug–metal interactions can selectively amplify restricted groups of vibrational modes, while the plasmonic metal substantially affects the resulting spectral response. Optimizing structures is an important step in using DFT, as the starting geometries involve one or more direct interactions between the psychoactive substance and the substrate [82,83,84]. Gold colloidal systems showed particularly favorable behavior in this case, demonstrating that selecting the metal cannot be reduced to maximizing the electromagnetic enhancement factor. Instead, the electronic interaction and adsorption geometry of the psychoactive molecule at the metal surface may determine which structural information is preferentially enhanced. Therefore, enhancement factors and detection limits reported for different SERS substrates are not necessarily directly comparable. In addition to substrate composition and morphology, these values depend on experimental parameters such as excitation wavelength and laser power, acquisition time, aggregation state, analyte adsorption and concentration protocol, and spectral processing. Furthermore, enhancement factors may be calculated using different definitions and reference conditions. Consequently, this study interprets the numerical values discussed below within the experimental conditions of the individual studies rather than using them independently to rank substrate performance.

Considerable efforts have therefore focused on engineering plasmonic metal nanostructures that maximize analyte adsorption and electromagnetic enhancement. Gold nanorods, gold-coated silicon nano-cone arrays, Au@Ag core–shell nanostructures, bimetallic silver-coated gold nanostars, and other nanostructured substrates have all demonstrated appropriate performance for the detection of psychoactive compounds, including cocaine, heroin, morphine, codeine, and promethazine [60,61,62]. These architectures generate numerous plasmonic “hot spots” that significantly increase Raman intensity, enabling rapid analysis with little or no sample preparation and detection limits ranging from the low nanogram to picogram per milliliter level. The use of solid plasmonic substrates further improves structural stability and measurement reproducibility compared with conventional colloidal nanoparticles, making them particularly attractive for portable forensic instrumentation [26,60]. In the paper by Hakonen and coworkers, commercially available Au and Ag nanopillar substrates are combined with handheld Raman instrumentation for in-field forensic analysis [27]. The flexible nanopillar architecture favors the formation of intense hot spots in the gaps between neighboring Ag caps, with calculated electromagnetic enhancement factors on the order of 10^10^ and experimentally determined average enhancement above 10^8^. Amphetamine could consequently be detected together with other forensic threat substances using commercially available substrates and portable instrumentation, demonstrating that SERS need not remain restricted to custom laboratory platforms. Reproducibility is particularly important when translating SERS to routine forensic screening. It should be evaluated at several levels, including variation between measurement positions on the same substrate (inter-spot), between independently prepared substrates (inter-substrate), and between different fabrication batches (inter-batch). Colloidal systems may show additional variability because SERS intensity depends strongly on nanoparticle aggregation, mixing conditions, ionic strength, and the resulting hot-spot distribution. Solid fabricated or commercial substrates can provide greater control over plasmonic architecture and measurement position; however, their forensic applicability still requires demonstration of reproducibility across substrates and production batches. These parameters are not consistently reported in the literature, which limits direct assessment of whether many proposed SERS platforms meet the reproducibility required for routine forensic screening.

Colloidal systems provide additional possibilities because nanoparticle aggregation can itself be manipulated to control the distribution and density of electromagnetic hot spots. This principle has recently been exploited for synthetic cathinones, a particularly challenging group of NPSs because small modifications of the common cathinone scaffold produce numerous closely related analogs [85]. Citrate-reduced Ag nanospheres combined with KBr were found to provide well-resolved SERS spectra for six synthetic cathinones, with KBr-induced aggregation increasing nanoparticle cluster size and the number of high-field regions. Importantly, the method was developed specifically for seized-drug screening and required less than one minute for analysis, illustrating the forensic advantage of controlling metal nanoparticle aggregation rather than relying only on the intrinsic properties of an isolated plasmonic particle. The study also showed that substrate selection depends on the analyte: several Au and Ag systems and different aggregating salts were evaluated before citrate-reduced Ag nanoparticles/KBr were identified as the most suitable combination.

Beyond substrate design, the adsorption behavior of psychoactive compounds on metallic nanostructures has emerged as another important factor governing SERS performance. Cocaine and its primary metabolite, benzoylecgonine, readily adsorb onto gold nanorods, enabling sensitive detection in latent fingerprints and oral fluid, whereas analyte-induced modification of silver nanoparticle assemblies has been demonstrated for berberine, showing that adsorption may actively alter the physicochemical properties of the plasmonic substrate itself. These observations show that metal–drug interactions in SERS extend beyond simple signal enhancement and involve dynamic processes at the metal–solution interface that influence spectral intensity, reproducibility, and concentration dependence [26,86]. This consideration becomes especially important for biological matrices, where endogenous components can compete for the metal surface or modify nanoparticle aggregation. A recent Au@Ag nanoparticle approach for psilocybin and its active metabolite, psilocin, for example, demonstrated extremely low detection limits of 1.11 × 10^−10^ and 6.75 × 10^−12^ M, respectively, with an enhancement factor of approximately 1.14 × 10^5^ [63]. The selected bimetallic core–shell particles containing Au and Ag exhibit synergistic effects of both metals, are also easy to prepare, show high stability, and have remarkable enhancement efficiency. The closely related compounds could nevertheless be differentiated through their characteristic SERS bands, demonstrating the molecular selectivity retained after plasmonic amplification [63]. Importantly, the study also illustrated a practical limitation of colloidal metal substrates: undiluted and two-fold diluted urine caused severe matrix-induced nanoparticle aggregation and suppressed the analytical signal, whereas ten-fold dilution effectively reduced this interference. Thus, control of the metal–sample interface can be as important as maximizing the nominal enhancement factor when SERS is transferred from standards to forensic or toxicological specimens.

The combination of metal-based SERS substrates with chemometric analysis and machine-learning algorithms further expands their forensic potential by enabling reliable discrimination among structurally related psychoactive compounds and quantitative analysis in complex matrices [87,88,89,90]. Recent studies have successfully integrated principal component analysis, orthogonal partial least-squares discriminant analysis, and data-enhancement algorithms with SERS measurements to distinguish illicit drugs in oral fluid, urine, latent fingerprints, and seized materials while maintaining rapid, portable, and non-destructive analysis [60,61]. However, high classification accuracy does not necessarily demonstrate generalizable forensic performance. SERS datasets often contain multiple spectra from the same sample or substrate, and random spectrum-level splitting between training and test sets can yield non-independent observations and inflate classification performance. More rigorous evaluation should therefore employ sample-level rather than spectrum-level splitting, independent test sets, and, where possible, external validation using different substrate batches, measurement sessions, instruments, and realistic sample matrices [91,92,93]. Examination of the spectral variables contributing to classification is also important to establish that discrimination originates from analyte-related spectral information rather than substrate, background, or preprocessing artifacts. These considerations are particularly important for translating promising SERS–machine-learning combinations from controlled datasets to routine forensic screening.

In general, SERS combines high analytical sensitivity with molecularly informative vibrational spectra, rapid measurement, and compatibility with portable Raman instrumentation, making it particularly attractive for forensic screening. Its principal limitations arise from substrate-to-substrate reproducibility, dependence on analyte adsorption and nanoparticle aggregation, matrix effects, and the need for standardized acquisition and data-processing protocols. Although engineered solid substrates and chemometric analysis can improve robustness and discrimination, reproducible performance across substrate batches, instruments, and authentic forensic samples remains essential for routine implementation.

## 5. Electrochemical Sensing Based on Metal Interactions

Metal-containing electrode modifiers play several complementary roles in the electrochemical analysis of psychoactive compounds. Importantly, the presence of a metal-containing component does not necessarily indicate a specific metal–analyte interaction. Mechanistically, these systems can involve direct electrochemistry of the analyte at a metal-modified electrode, coordination-based recognition at a metal center, metal-mediated electrocatalysis, indirect sensing through a metal-based redox probe, or enhancement of electrode properties such as conductivity, electroactive surface area, and analyte adsorption. In the latter case, the metal-containing material primarily facilitates signal generation rather than providing chemically specific analyte recognition. Coordination complexes may act as reversible redox mediators, provide binding sites for analyte accumulation, or stabilize intermediates formed during drug oxidation or reduction [94,95,96]. Metal and mixed-metal oxides, metal–organic frameworks, and metal–carbon composites additionally increase the electroactive surface area, lower charge-transfer resistance, and catalyze electron-transfer reactions that are weak or poorly resolved at unmodified electrodes. These effects are particularly important for psychoactive compounds because their electrochemical responses frequently involve oxidation of amino, phenolic, or phenothiazine groups and may be affected by high overpotentials, electrode fouling, or overlapping signals from adulterants [94]. Recent developments in ephedrine analysis similarly demonstrate that carbon–metal oxide composites, nanostructured materials, conducting polymers, and molecular-recognition layers can substantially improve sensitivity and selectivity while supporting miniaturized and portable detection systems [95].

The clearest examples of coordination-mediated electrochemical detection have been reported for cocaine. In cobalt hexacyanoferrate-modified electrodes, cocaine interacts reversibly with Co ions within the inorganic film, partially passivating the electrode surface and producing a concentration-dependent decrease in the original redox current [64]. This mechanism was used for the analysis of confiscated samples and was subsequently adapted to screen-printed electrodes and portable potentiostats, illustrating how a specific metal–drug interaction can be converted directly into a field-compatible analytical signal [64,97]. Schiff-base complexes of Mn(II), Fe(II), Ni(II), Cu(II), Co(II), and uranyl ions have also been incorporated into carbon-paste or platinum electrodes [65,98]. In the Mn(II) system, reduction of the cocaine carbonyl group was proposed to generate a radical intermediate stabilized through interaction with the metal d orbitals, whereas the Co(II)–salen system promoted cocaine oxidation through a coupled redox process involving oxidation of both cocaine and the metal complex [65,98]. Density functional calculations supported simultaneous conversion of [Co^II^(salen)] into a higher oxidation state and two-electron oxidation of cocaine to an iminium intermediate, followed by hydration and *N*-dealkylation [98]. The distinct oxidation potentials of cocaine, lidocaine, procaine, and theobromine further enabled chemometric discrimination of the target drug from common interferents, emphasizing that the coordination compound acts not only as an electrocatalyst but also as a chemically responsive recognition layer.

Nanostructured metal and mixed-metal oxides primarily enhance sensing via electrocatalysis rather than stoichiometric complex formation. Iron molybdate nanoplates increased the oxidation current of promethazine while shifting its response to a lower anodic potential relative to a bare glassy-carbon electrode [66]. SmCoO_3_ nanoparticles produced a similar effect by facilitating charge transfer between promethazine and the electrode surface, while NiMoO_4_/CoMoO_4_ nanoflakes combined a large electroactive area with synergistic contributions from the two transition-metal components for chlorpromazine determination [10,96]. These platforms provided sensitive responses in pharmaceutical, biological, water, and wastewater samples [10,66,96]. A related strategy was employed for opioids using a screen-printed carbon electrode bearing three-dimensional Au structures coated with NiO_x_. Gold improved conductivity and surface area, whereas the nickel oxide layer enhanced electrocatalytic oxidation, producing separate peaks for morphine and fentanyl and therefore permitting their simultaneous determination [99]. Earlier morphine sensors likewise employed copper complexes and graphene-based coatings, in which Cu(II) served as an electroactive center, and the carbon material increased adsorption and electron transfer; such systems lowered the oxidation potentials of both the phenolic and tertiary amine sites of morphine and reduced surface fouling [94].

Porous coordination materials offer an additional advantage by combining analyte adsorption with accessible redox-active metal sites. A Cu–Co 2-methylimidazolate MOF anchored on carbon nanofibers allowed dopamine and tyramine to diffuse through a hydrophilic, high-area matrix toward Cu and Co centers, where faster electron transfer and electrocatalytic oxidation generated resolved analytical signals [67]. Functional groups within the framework additionally acted as adsorption sites, while the carbon nanofibers provided conductivity and mechanical stability. Selectivity may also be introduced through molecularly imprinted polymer films. In the reported tyramine sensor, recognition cavities controlled access of a ferri/ferrocyanide redox probe to the electrode through a concentration-dependent “gate effect.” Although the analyte was not oxidized directly at a metal center, the Fe(CN)_6_^3−^/^4−^ couple translated selective tyramine binding into a voltammetric or impedance response [100]. These examples demonstrate that metal-containing components may participate at different stages of electrochemical sensing: through direct coordination of the analyte, electrocatalytic conversion at a metal site, mediation by a metal-based redox probe, or improvement of conductivity, surface area, and adsorption within a hybrid material. Importantly, only the former mechanisms necessarily involve a chemically specific interaction between the metal center and the analyte.

Electrochemical platforms offer advantages of high sensitivity, relatively low instrumental cost, rapid analysis, miniaturization, and compatibility with disposable screen-printed electrodes and portable potentiostats. However, overlapping redox responses, electrode fouling, matrix components, and variability in modified electrode surfaces can still affect analytical performance. Moreover, increased current from improved conductivity, surface area, or electrocatalysis should not be interpreted as enhanced molecular selectivity. Consequently, their greatest potential for forensic screening will depend on combining robust electrode fabrication with chemically selective recognition and validation in realistic samples.

## 6. Conclusions and Future Prospects

Interactions between psychoactive compounds and metals provide a remarkably diverse foundation for analytical detection, extending far beyond conventional coordination-complex formation. As this review shows, metals may participate directly in molecular recognition through coordination and ion association, mediate oxidation–reduction reactions, generate or modulate optical signals, promote adsorption and charge transfer at metallic surfaces, or catalyze electrochemical transformations. The analytical role of the metal, therefore, depends strongly on the sensing system’s architecture. Classical colorimetric reactions represent the simplest implementation of these interactions, whereas luminescent nanomaterials, plasmonic SERS substrates, metal–organic frameworks, and metal-modified electrodes increasingly integrate several functions, including recognition, preconcentration, signal generation, and amplification, within a single analytical platform.

From a forensic perspective, this evolution is particularly important because metal-based systems can address some of the limitations associated with conventional presumptive testing. The cobalt thiocyanate test for cocaine illustrates both sides of this problem: metal coordination can provide an immediate and easily interpreted response, but limited chemical selectivity restricts the evidential significance of the result. More advanced approaches increasingly replace a simple positive/negative response with richer analytical information. Photoluminescence spectra of analyte-specific coordination clusters, characteristic SERS fingerprints, and electrochemical profiles recorded at metal-modified electrodes can provide multiple analyte-dependent variables, thereby improving discrimination among structurally related substances. Incorporating chemometric and machine-learning methods may further exploit these multidimensional signals, particularly when distinguishing closely related psychoactive substances, cutting agents, and complex mixtures is required.

Among the approaches discussed, SERS currently provides a particularly attractive combination of molecular specificity, high sensitivity, rapid analysis, and compatibility with portable instrumentation. Future development should therefore emphasize reproducible, stable substrates; controlled surface chemistry; standardized measurement protocols; and validation across realistic concentrations and matrices, rather than pursuing progressively lower detection limits as the primary measure of analytical performance.

Luminescent and electrochemical platforms offer complementary opportunities. Multimetallic nanoclusters, quantum dots, MOFs, and hybrid magnetic–fluorescent systems demonstrate how the composition and surface chemistry of a metal-containing material can be engineered to control analyte accessibility, charge transfer, and signal modulation. Particularly promising future platforms may therefore be those in which the metal-containing component is deliberately designed to combine selective recognition with signal amplification, rather than serving merely as a high-surface-area electrode modifier or generic nanomaterial.

The principal challenge for future research is therefore not demonstrating additional highly sensitive metal-based sensors, but translating them from proof-of-concept experiments to operational forensic practice. Several barriers remain particularly important. First, many reported systems have been evaluated primarily using pure standards or relatively simple matrices, whereas forensic samples may contain adulterants, metabolites, pharmaceuticals, salts, proteins, and structurally related psychoactive substances that can compete for coordination or adsorption sites and modify optical or electrochemical responses. The origin of matrix interference depends on the platform: competing coordination or redox reactions can affect colorimetric and spectrophotometric methods; endogenous fluorophores and quenchers may alter luminescence responses; proteins and salts can modify analyte adsorption or nanoparticle aggregation in SERS; and electrochemical methods may be affected by competing redox-active species and electrode fouling. Second, reproducibility remains a critical issue, particularly for nanostructured materials whose analytical response may depend strongly on substrate preparation, surface chemistry, aggregation state, and storage conditions. Third, the lack of standardized measurement and validation protocols complicates comparison between independently developed platforms. Finally, relatively few methods have undergone extensive validation using authentic seized materials or biological specimens. Future studies should therefore prioritize blinded analyses of realistic samples, inter-day and inter-batch reproducibility, long-term substrate stability, interference studies involving realistic drug mixtures, and direct comparison with established confirmatory techniques.

Metal-based detection methods are unlikely to replace chromatographic–mass spectrometric techniques for definitive forensic identification, as shown in Figure 2. Their greatest potential lies instead in providing a new generation of rapid, inexpensive, portable screening tools that generate substantially more chemically informative responses than traditional presumptive tests. Future development will likely involve greater integration of engineered metal-containing materials with handheld Raman systems, screen-printed electrodes and portable potentiostats, miniaturized sample preparation, and chemometric or machine-learning-assisted interpretation. Particularly promising directions include multifunctional materials that combine analyte recognition, preconcentration, and signal amplification within a single platform, as well as disposable sensing devices capable of decentralized analysis of seized materials and biological specimens. Such systems could support on-site drug screening, rapid prioritization of samples for confirmatory analysis, and the detection of newly emerging psychoactive substances for which conventional screening libraries may initially be limited. However, successful implementation will require not only improved analytical performance but also robust validation, standardized data acquisition, interpretable classification models, and compatibility with established forensic workflows. Future progress will therefore depend on treating the chemistry of the metal–analyte interface not merely as a means of maximizing analytical sensitivity, but as a source of molecular information that can be rationally understood, optimized, and validated for forensic decision-making.

## Figures and Tables

**Figure 1:**
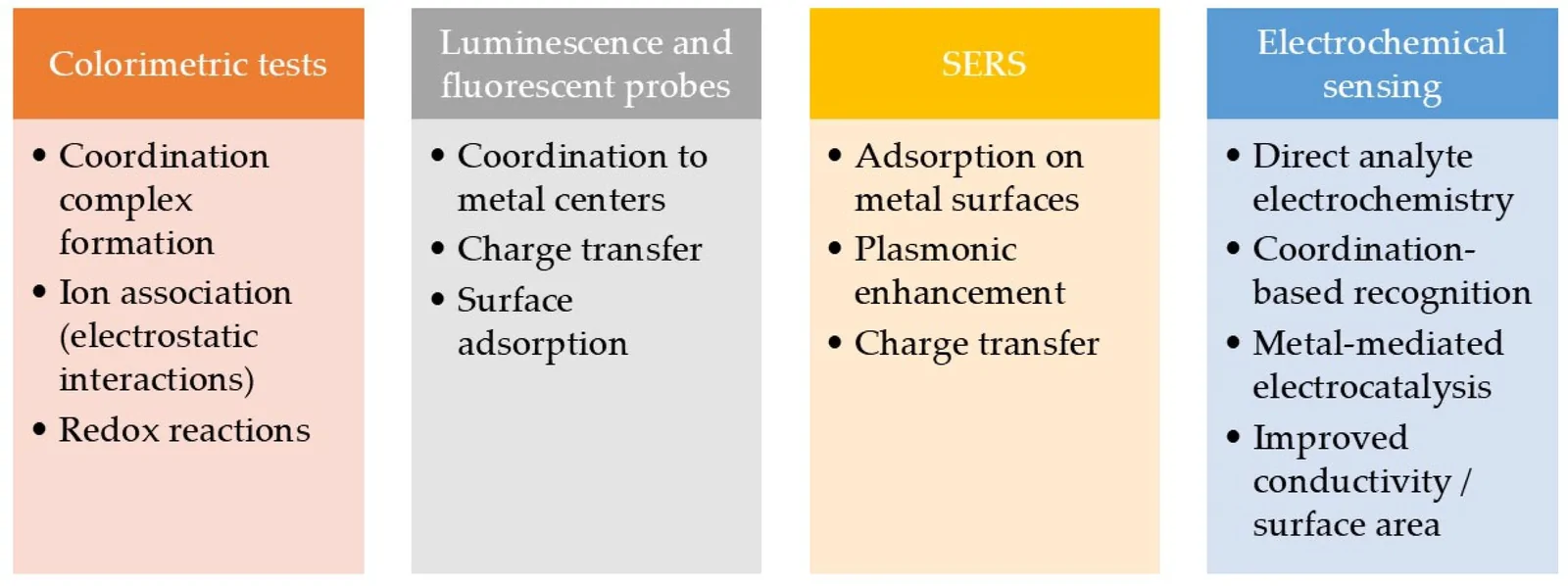
Major metal–analyte interaction mechanisms involved in detecting psychoactive compounds.

**Figure 2:**
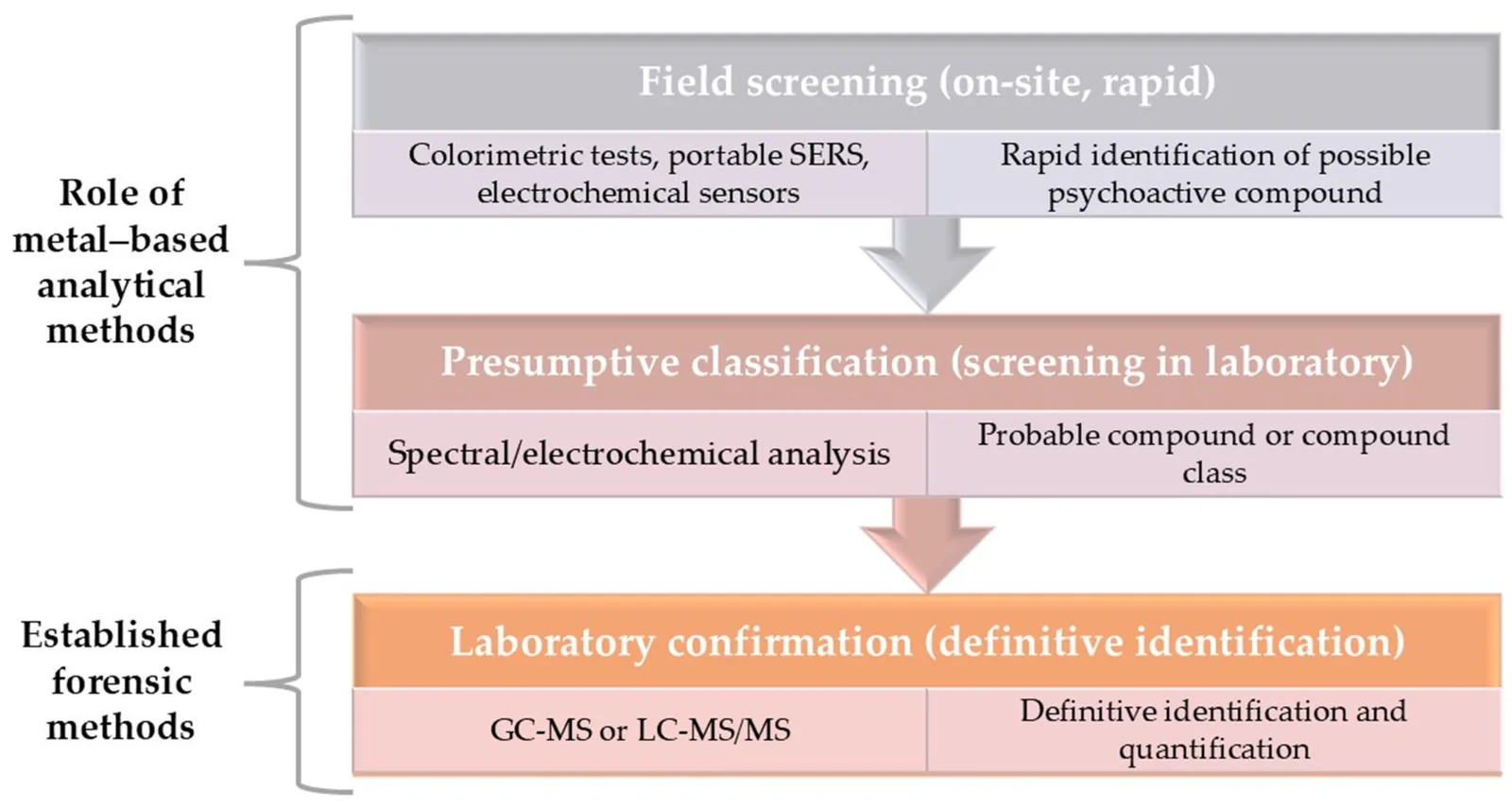
Proposed workflow for the integration of metal-based analytical methods into forensic analysis of psychoactive compounds.

**Table 1:** Analytical performance and forensic applicability of selected metal-based methods for the detection of psychoactive compounds.

Compound(s)	Metal/Material	Metal–Analyte Role/Mechanism	Technique	Matrix/Real Sample Verification	Parameters	Interfering Substances (NS—Not Specified, NO—Not Observed)	Reference
Cocaine	Cobalt(II) thiocyanate	Coordination	UV–VIS	Aqueous solution/forensic sample	/	Fentanyl	Haddoub et al., 2011 [9]
Ketamine hydrochloride	Cobalt(II) thiocyanate	Color change, mechanism not shown	UV–VIS	Aqueous solution	LOD: 5 mg mL^−1^	Hydrocodone, zolpidem	Morris, 2007 [36]
Morphine	Ceric ammonium sulfate	Ce(IV) reduction to Ce(III)	UV–VIS and spectrofluorimetry	Aqueous solution/tablets, ampoules, spiked biological fluids	Linear range: 1.6–8 µg mL^−1^ (UV–VIS), 1.6–12.8 µg mL^−1^ (spectrofluorimetry) LOD 0.0142 µg mL^−1^ (UV–VIS) and 0.0526 µg mL^−1^ (spectrofluorimetry)	NS	El-Didamony and Ali, 2013 [25]
Chlorpromazine hydrochloride	Ceric ammonium sulfate	Ce(IV) reduction to Ce(III) in acidic medium	UV–VIS and spectrofluorimetry	Aqueous solution/tablets and capsules	Linear range: 0.05 to 1.3 µg mL^−1^ LOD: 0.036 µg mL^−1^ LQD: 0.119 µg mL^−1^	NO	Mohamed et al., 2005 [42]
Ketamine	Cu(II) and BODIPY-Phen conjugate	Cu(II) reduction to Cu(I) and coordination to BODIPY-Phen conjugate	Spectrofluorimetry	Soft drinks and alcoholic beverages	Linear range: 1–16 µM LOD: 2.88 µM (ketamine)	NO	Hernández-Contreras et al., 2024 [43]
Synthetic cathinones	Cu(II)-2,9-dimethyl-1,10-phenanthroline	Cu(II) reduction to Cu(I) and change in color	UV–VIS	Aqueous solution	LOD: 40 µg (4-methylmethcathinone hydrochloride)	NO	Philp et al., 2016 [45]
Promazine hydrochloride	K_2_Cr_2_O_7_ in acidic medium	Oxidation of promazine hydrochloride to promazine sulphoxide	UV–VIS	Aqueous solution/tablets	Linearity range: 100–700 µg mL^−1^	NO	Misiuk et al., 2002 [46]
Papaverine	Acidic solution of Ce(IV)	Oxidation of papaverine and reduction of Cu(IV) to Ce(III), enhancement of CL signal of sulfite-cerium(IV)	Chemiluminescence	Aqueous solution/urine, and serum	Linear range: 1 × 10^−7^–1 × 10^−5^ M LOD: 8.7 × 10^−8^ M	Narcotine	Zhang et al., 2004 [23]
Bromazepam	Eu(III)	Eu(III)-bromazepam complex formation, luminescence	Spectrofluorimetry	Ethanol and acetonitrile solution/tablets	Linear range: 2.3 × 10^−8^–6.2 × 10^−7^ M LOD: 3 × 10^−9^ M LOQ: 1.2 × 10^−8^ M	NS	Attia, 2009 [47]
Promazine, chlorpromazine, promethazine	Potassium ferricyanide, ammonium reineckate and sodium cobaltinitrite	Ion-association	Precipitation	Water solution	Not specified	NS	El-Ansary et al., 2001 [39]
Ephedrine	Co(II)	Complex formation	UV–VIS	Extraction by chloroform/pharmaceutical preparations	LOD: 0.99 ppm Relative standard deviation: 0.303% Sandel’s sensitivity: 0.0135	NS	Alminshid et al., 2022 [24]
GBH	Iridium(III) complex with bipyridine and phenanthroline	Luminescence quenching	Spectrofluorimetry	Aqueous solution/spiked real-life beverages	Minimum GBH concentration inducing 30% quenching: 0.15 mg mL^−1^.	NO	Wang et al., 2017 [48]
GBH	BODIPY-Fe(III)	Luminescence quenching and absorption change	Spectrofluorimetry	Aqueous solution/spiked real-life beverages	LOD: 0.25 µg mL^−1^ (absorption) and 0.01 µg mL^−1^ (emission)	NO	Ryu and Kim, 2022 [49]
GBH	Cu(II)-complex with tetradentate ligand and coumarin dye	Displacement between coumarin and GBH	Spectrofluorimetry	Aqueous solution/spiked real-life beverages	LOD: 0.03 µM in MES buffer solution and 0.1 µM in soft and alcoholic drinks	NO	Garrido et al., 2023 [50]
Cocaine and metabolites	Mn-doped ZnS quantum dots with molecularly imprinted polymer	Electron-transfer-induced quenching of fluorescence by cocaine	Spectrofluorimetry	Aqueous solution/urine samples from cocaine users	Linear quenching up to 1 mg L^−1^	NO	Chantada-Vázquez et al., 2016 [51]
Methamphetamine	Adrenaline polymer on Co(II) quantum dots	Specific binding of methamphetamine to the surface and formation of a non-fluorescent complex	Spectrofluorimetry	Artificial urine	Linear range: 67–469 nM, LOD: 23 nm Recovery rate: 96.2–102.1%	NO	Yin et al., 2026 [52]
Methamphetamine	Molecularly imprinted EDTA-Cu(II) quantum dots	Specific adsorption and enhancement of fluorescence intensity, not selective response	Spectrofluorimetry	Artificial urine	Linear range: 53–371 nM LOD: 20.6 nM Recovery rate: 97.2–104.4%	NO	Zhu et al., 2026 [40]
3,4-methylenedioxymethylamphetamine (MDMA)	AuZnFeSeS alloyed quantum dots and spinel NiCeFe_3_O_4_ with anti-MDMA antibody	Specific adsorption and signal quenching, NiCeFe_3_Oa was used for signal amplification	Spectrofluorimetry	Urine sample	Linear range: 0.05–50.000 nM LOD: 0.02 nM	NO	Adegoke and Daeid, 2023 [53]
Morphine	Metal–organic framework with Cr(III) nanoparticles	Signal enhancement due to interactions of morphine with the sensor	Spectrofluorimetry	Spiked urine and serum	Linear range: 0.1–350 nM LOD: 0.167 nM LQD: 0.443 nM	NO	Alhaddad and Sheta, 2020 [54]
Ketamine, morphine, and heroin	Levofloxacin-derived carbon dots and UiO-66-NH_2_	Fluorescence enhancement due to combined effects of constituents, hydrogen bonding, and π-π interactions	Spectrofluorimetry	Tap water and wastewater samples	Linear range: 1–200 ng L^−1^ (ketamine) and 1–100 ng L^−1^ (morphine and heroin)	NO	Mohammadi et al., 2026 [55]
Morphine	Au nanoparticles with fluorescein	Interactions between morphine and Au nanoparticles lead to the release of fluorescein and signal enhancement	Spectrofluorimetry	Spiked urine and serum/sweat, urine, and other biological fluids	Linear range: 0.0046–9.97 pM and 17.4–29.9 nM LOD: 53 pM, 67.1 pM (serum), 62.5 pM (urine)	NO	Nebu et al., 2018 [56]
Methamphetamine	Captopril-modified Y_2_O_3_:Eu^3+^ nanoparticles	Fluorescence emission enhancement due to interactions of methamphetamine and sensor	Spectrofluorimetry	Spiker urine	Linear range: 0.2–30 µM LOD: 0.14 µM	NS	Goudarzy et al., 2022 [57]
Amphetamine and morphine	Trimetallic (Au, Ag, Cd) nanoparticles stabilized by lysosome, glutathione, and 4-mercaptopropionic acid	Interactions between analytes and metal clusters that depend on the used modifiers, fluorescence enhancement	Spectrofluorimetry	Aqueous solution	LOD: 4.44 µM (AuAgCd-Lyz) and 22.32 µM (AuAgCd-GSH) for amphetamine; 2.16 µM (AuAgCd-Lyz) and 4.97 µM (AuAgCd-GSH) for morphine	NS	Paria et al., 2026 [58]
Cocaine, phencyclidine, fentanyl, opiates, piperazine-based designer drugs	Cu(I)	Photoluminescence due to formation of organometallic nanoclusters	Spectrofluorimetry	Aqueous solution	LOD: 0.150 mg (phencyclidine), 1 mg (cocaine), 0.015 mg (piperazine), 0.150 mg (morphine sulfate)	NO	Nash et al., 2018 [59]
Promethazine hydrochloride	Au@Ag double-ended nano-pencils	Electromagnetic field enhancement	SERS	Aqueous solution	LOD: 0.1 ppb	NO	Bao et al., 2025 [60]
Cocaine	Gold nanorods	Electromagnetic field enhancement	SERS	Oral fluid	LOD: 10 ng mL^−1^	NO	D’Elia et al., 2018 [61]
Cocaine and heroin	Bimetallic gold–silver nanostars	Electromagnetic field enhancement	SERS	Aqueous solution/urine	LOD: 10 ng mL^−1^ (cocaine) 100 ng mL^−1^ (heroin)	NO	Atta and Vo-Dinh, 2023 [62]
Psilocybin and psilocin	Silver-coated gold nanoparticles (Au@AgNPs)	Electromagnetic field enhancement	SERS	Urine	LOD: 1.11 × 10^−9^ M (psilocybin) 6.75 × 10^−9^ M (psilocin)	NO	Chen et al., 2026 [63]
Cocaine	Platinum disk electrode chemically modified with cobalt-hexacyanoferrate	Reversible interaction with the film	Voltammetry	Aqueous solution/confiscated samples	Linear range: 2.4 × 10^−4^–1.5 × 10^−3^ M LOD: 1.4 × 10^−4^ M	NO	Oiye et al., 2009 [64]
Cocaine hydrochloride	Carbon paste chemically modified with N,N′-ethylene-bis-(salicylideneiminato) manganese(II)	Electronic exchange between cocaine hydrochloride and the electrode surface	Voltammetry	Aqueous solution	Linear range: 1–100 µM LOD: 0.9349 µM LOQ: 3.11µM	NO	Castro et al., 2020 [65]
Promethazine hydrochloride	Iron molybdate nanoplates	Electrochemical oxidation	Voltammetry	Aqueous solution/commercial tablets	Linear range: 0.01–68.65 µM LOD: 0.01 µM	NO	Arumugam et al., 2020 [66]
Dopamine and tyramine	Bimetallic copper–cobalt MOFs	Adsorption and electronic exchange	Voltammetry	Aqueous solution/food beverages	Linear range: 5–800 µM LOD: 44.2 nM (dopamine) and 52.7 nM (tyramine)	NO	Ahmad et al., 2023 [67]

## Data Availability

The data are contained in this article. Further inquiries can be directed to the author.
